# Intermittent and mild cold stimulation enhances immune function of broilers via co-regulation of CIRP and TRPM8 on NF-κB and MAPK signaling pathways

**DOI:** 10.1016/j.psj.2024.103984

**Published:** 2024-06-17

**Authors:** Lu Xing, Haochen Li, Deyang Miao, Haidong Wei, Shijie Zhang, Qiang Xue, Hongyu Wang, Jianhong Li

**Affiliations:** ⁎ College of Life Science, Northeast Agricultural University, Harbin, 150030, China; † Key Laboratory of Chicken Genetics and Breeding, Ministry of Agriculture and Rural Affairs, Harbin, 150030, China

**Keywords:** broiler heart, cold stimulation, CIRP, TRPM8, immunity

## Abstract

Improving immune function is an important indicator for establishing cold adaptation in broilers. In the study, to explore the effects and molecular mechanisms of intermittent and mild cold stimulation (**IMCS**) on the immune function of broilers, CIRP and TRPM8, induced by cold stimulation, as well as the NF-κB and MAPK pathways which play an important role in immune response, were selected to investigate. A total of 192 one-day-old broilers (Ross 308) were selected and randomly divided into the control group (**CC**) and the cold stimulation group (**CS**). The broilers in CC were raised at normal feeding temperature from d 1 to 43, while the broilers in CS were subjected to cold stimulation from day 15 to 35, with a temperature 3 °C below that of the CC group for 5 h, at 1 d intervals. The results showed that IMCS had little effect on the broiler hearts, and the myocardial structure was not damaged. On d 22, IMCS significantly increased the mRNA levels of *CIRP, TRPM8, P65, P38, COX-2, TNF-α, IFN- γ, IL-6, IL-10*, and the protein levels of CIRP, P65, P38, IL-1β and iNOS in the hearts, and the levels of CIRP and all cytokines in the serum (*P* ≤ 0.05). The mRNA and protein levels of IκB-α were significantly reduced (*P* ≤ 0.05). On d 36, the mRNA levels of *TRPM8, P65, ERK,* and *IL-10* in the hearts and the content of COX-2 in the serum in CS were increased significantly (*P* ≤ 0.05), while the mRNA levels of *IκB-α, P38,* and *IL-1β* were decreased significantly (*P* ≤ 0.05). On d 43, IMCS significantly upregulated the mRNA levels of *TRPM8, IFN- γ, IL-4, IL-6, IL-10*, and the protein levels of IκB-α, P38, and the levels of iNOS, TNF-α, IL6 and IL10 in the serum (*P* ≤ 0.05); whereas it significantly downregulated *CIRP, JNK, P38, iNOS, TNF-α* mRNA levels, and CIRP, P65, ERK, JNK, IL1β and iNOS protein levels (*P* ≤ 0.05). Therefore, IMCS can enhance broiler immune function through co-regulation of CIRP and TRPM8 on the NF-κB and MAPK pathways, which facilitate the cold adaptation in broilers.

## INTRODUCTION

The adverse effects of cold stress in alpine regions seriously affect the welfare and health of livestock and poultry, and restrict the development of animal husbandry. Animal behavior, production performance, immune function, energy metabolism, and antioxidant system levels can all be negatively affected by acute or long-term cold stress (Li et al., 2017; Xie et al., 2017; Henrikson et al., 2018; Liu et al., 2022; Gong et al., 2023). Under long-term cold stress, mice release more pro-inflammatory cytokines, leading to an increased incidence of neuroinflammation (Xu et al., 2020). Acute cold stress can disrupt the immune balance of broilers and weaken non-specific immunity (Wei et al., 2018). Cold stress can also lead to a decrease in the number of lymphocytes in laying hens, degradation of the lymphatic organs, and thus suppression of immune function (Spinu and Degen, 1993). However, appropriate cold stimulation can help the body adapt to low-temperature environments, enhance its temperature regulation, antioxidant, and immune abilities, as well as stress resistance to prevent cold injury (Wei et al., 2024; Xing et al., 2023). Rats exposed to cold during the neonatal period show enhanced cold resistance (Agrawal et al., 2011). Xu et al. (1992) found that the cell-mediated immune function of mice was significantly enhanced after being raised in a 2 °C environment for 2 wk. Similarly, cold stimulation training can improve the immune function and anti-inflammatory ability of broilers by increasing the expression of cytokines, while the immune imbalance caused by acute cold stress is alleviated (Su et al., 2018a; Su et al., 2020). Exposure to a 15°C environment at 3 and 4-days-old can enhance the cold tolerance of broilers, and mortality due to cold temperatures is significantly reduced (Shinder et al., 2007). At present, mitigating the negative effects of cold stress on animal husbandry is a topic of general interest.

The nuclear factor-kappa B (**NF-κB**) signaling pathway is closely related to immune responses (Smale, 2011). Activation of NF-κB can induce the overexpression of certain inflammatory cytokines, such as tumor necrosis factor-α (**TNF-α**), interleukin (**IL**) -1β, IL-8, inducible nitric oxide synthase (**iNOS**), and cyclooxygenase-2 (**COX-2**). Research has shown that long-term low-temperature exposure and acute cold stress can both induce the overexpression of inflammatory cytokines and an immune imbalance of broilers, which is mediated by NF-κB (Wei et al., 2018). Upon the appropriate cold stimulation, the cold injury in the ileum of broilers is relieved, and immune function is improved (Liu et al., 2022). This is mainly due to the decrease in inflammatory cytokine levels caused by the NF-κB signaling pathway, which prevents immune imbalance after cold stress. The mitogen activated protein kinases (**MAPKs**) signaling pathway, which involves the extracellular regulated protein kinases (**ERK**), P38, and c-Jun N-terminal kinase (**JNK**) signaling pathways, can be activated by external stimuli (Nakahara et al., 2006). The activation of the MAPK signaling pathway can trigger humoral immunity, and it can promote the upregulation of TNF-α and IL-6 levels and the regulation of inflammation (Drube et al., 2016). However, the secretion of anti-inflammatory cytokines (IL-4 and IL-10) can trigger cellular and humoral immune responses, and weaken inflammatory responses (Su et al., 2018b; Shi et al., 2019). A study has shown that phosphorylation of the ERK pathway could upregulate the expression of thermogenic genes in the brown adipose tissue (**BAT**) of mice to promote cold adaptation (Lu et al., 2019).

The cold inducible RNA-binding protein (**CIRP**) is one of stress proteins released after mild hypothermia stimulation, and protects the body by regulating the cell cycle, inhibiting apoptosis and preventing cell senescence (Saito et al., 2010; Zhang et al., 2015). Under mild cold stimulation, the expression level of CIRP is gradually increased with a decrease in temperature. It can bind to toll-like receptor 4 (**TLR4**) and participate in the inflammatory response through the NF-κB pathway, where it is a positive regulatory molecule of the NF-κB signaling pathway (Chen et al., 2016). The upregulation of CIRP can increase the mRNA expression levels of IL-8, IL-1 β and TNF-α, which is mediated by the NF-κB signaling pathway (Gallouzi et al., 2013). Also, CIRP can protect cells from apoptosis induced by TNF-α through the ERK signaling pathway, under low temperature exposure (Sakurai et al., 2006). Conversely, downregulation of CIRP can induce apoptosis of germ cells by activating the P38 and SAPK/JNK signaling pathways (Xia et al., 2012).

Transient receptor potential melastatin 8 (**TRPM8**) is a peripheral neural sensor, which can be activated by mild cold temperatures and exogenous molecules such as menthol. It plays an important role in maintaining the homeostasis of the intracellular and extracellular environment and regulating adaptation to the environment by perceiving external and internal stimuli and then mediating temperature regulation, cell differentiation, and apoptosis. Research has shown that mice with a TRPM8 expression deficiency have a reduced responsiveness to cold environments (Bautista et al., 2007). TRPM8 not only perceives low temperatures but also participates in the regulation of the inflammatory response under low-temperature environments, which is related to immune protection. Human osteoarthritic chondrocytes cultured with menthol can activate the expression of TRPM8, which can increase the expression of iNOS and IL-6 (Halonen et al., 2023). Wang et al. (2017) suggested that during cold stress, TRPM8 could inhibit the dissociation and nuclear translocation of NF-κB, and negatively regulate TNF-α expression in the hypothalamus of mice. In addition, activation of TRPM8 by low temperatures can inhibit the P38 pathway, thereby playing a therapeutic role in asthma (Zhang et al., 2016).

Appropriate cold stimulation can help the body adapt to cooler temperatures, but research on the molecular mechanisms of cold adaptation in poultry is scarce and the mechanism by which cold stimulation can enhance the immunity of broilers is not yet clear. This study aimed to elucidate the molecular mechanisms of cold adaptive responses in broilers. We analyzed that the regulatory effect of CIRP and TRPM8 on the NF-κB and MAPK signaling pathways and their impact on immune function during broiler exposure to intermittent and mild cold stimulation (**IMCS**). This study lays the foundation for enriching the theory of cold adaptation in broilers and also provides a scientific basis for the development of animal husbandry in cold regions using the principle of cold adaptation.

## MATERIALS AND METHODS

### Animal Care and Experimental Design

**Figure 1:**
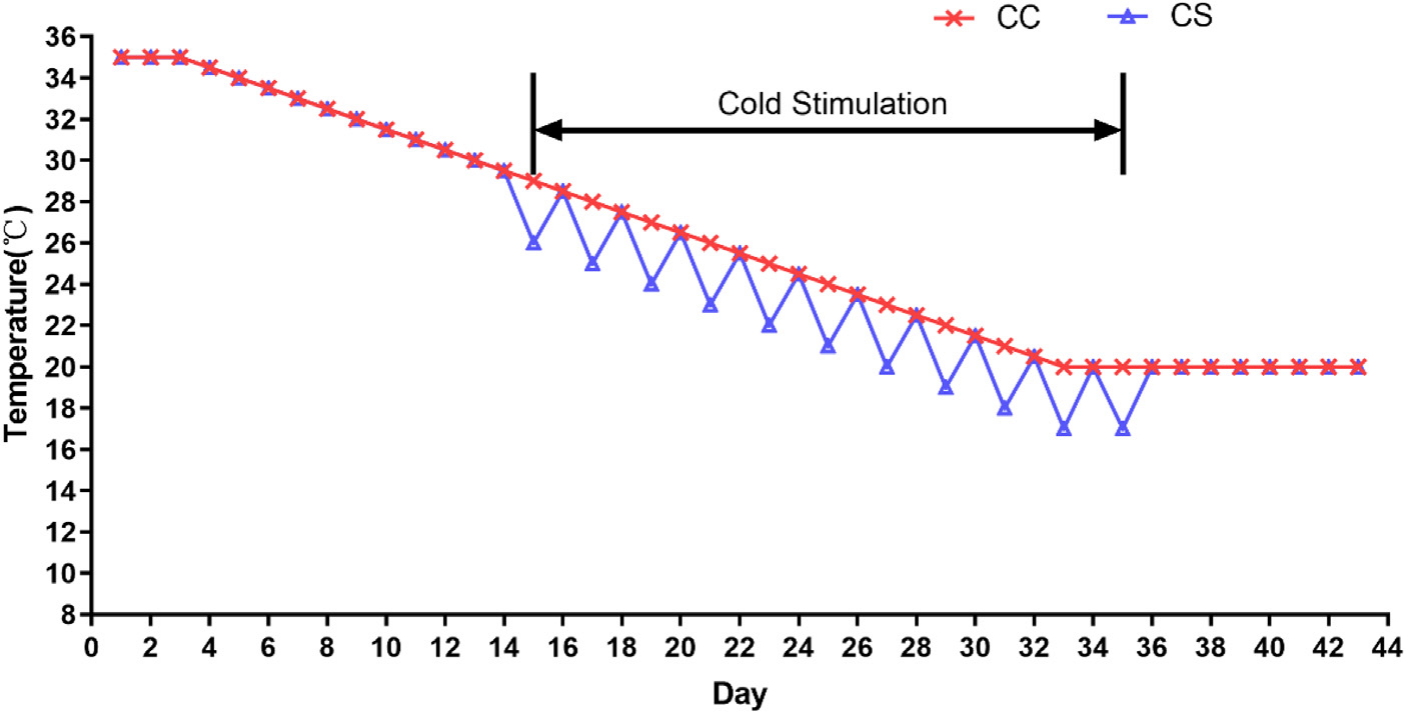
The specific feeding temperature in this study.

A total of 192 one-day-old Ross 308 male broilers were selected and randomly divided into two groups, each group included six replicates with 16 chickens per replicate. The feeding schedule was based on our previous study (Zhang et al., 2024). Briefly, the control group (**CC**) was raised at normal feeding temperature, which was 35 °C in the first 3 d, then the temperature was decreased by 1 °C every other day until reaching 20 °C at the age of 33 d. The cold stimulation group (**CS**) was subjected to intermittent and mild cold stimulation (**IMCS**). The chickens in CS group were raised at the same temperature as CC for 1 to 14 d, from 15 to 35 d, the temperature was 3 °C lower than the CC group every other day. Cold stimulation in CS group started from 9:30 to 14:30 every other day for a duration of 5 h. From 36 to 43 d, all birds in CC and CS groups were kept at an ambient temperature of 20 °C. The specific temperature is shown in Figure 1. The humidity of the rooms was kept at 60 to 70% from 1 to 14 d and 40 to 50% from 15 to 43 d. The light regime was 24 h light: 0 h dark on 1 to 3 d, and 23 h light: 1 h dark on 4 to 43 d. During the experiment, broilers were provided water and feed ad libitum. A commercial starter diet was provided for 1 to 21 d (metabolizable energy [**ME**] of 12.1 MJ/kg and crude protein [**CP**] of 21.0%) and a grower diet was given for 22 to 43 d (ME of 12.8 MJ/kg and CP of 19.0%) (Baishicheng Animal Husbandry, Harbin, China). All experiments and procedures performed in this study were approved and conducted according to the Institutional Animal Care and Use Committee of the Northeast Agricultural University (IACUCNEAU20150616).

On d 22, 36, and 43, one chicken was randomly selected from each replicate per group for euthanasia. Heart tissues were quickly removed and washed with normal saline. Heart tissues (1 cm × 1 cm × 1 cm) was cut and fixed in 4% paraformaldehyde, and the remaining tissues were rapidly transferred to liquid nitrogen for freezing, finally tissues were saved in -80 °C. The collected blood sample was stood at room temperature for 2 h, then centrifuged at 2000 × g for 10 minutes. Finally, the serum was sucked out, and kept at -20 °C.

### Examination Under Optical Microscope

The specific method was referenced to Li et al (Li et al., 2018). Heart tissues were taken out and soaked in 4% paraformaldehyde. Then tissues were dehydrated with different concentrations of ethanol, placed in xylene for transparent, and finally embedded in paraffin. A 5 μm thickness was then prepared and stained with hematoxylin and eosin (**H&E**). Finally, the piece was observed by an optical microscope (Leica, Germany).

### Elisa Analysis

The enzyme-linked immunosorbent assay (**ELISA**) was used to determine the levels of CIRP, iNOS, COX-2, TNF-α, IL-1β, IL-4, IL-6, IL-10 and interferon-γ (**IFN-γ**) in serum according to the manufacturer's instructions (Xinle, Shanghai, China).

### RNA Extraction and Reverse Transcription

Total RNA was extracted from hearts using Rnaiso plus (Takara, Dalian, China) according to the manufacturer's instructions. The RNA concentration and purity were detected and recorded. The ratio of OD260 to OD280 should be between 1.8 and 2.1. Complementary DNA (**cDNA**) was synthesized through reverse transcription according to the manufacturer's instructions (Toyobo, Japan).

### Quantitative Real-Time PCR Examination

The forward and reverse primers of target genes (*CIRP, TRPM8, IκB-α, P65, ERK, JNK, P38, iNOS, COX-2, TNF-α, IL-1β, IL-4, IL-6, IL-10* and *IFN-γ*) and reference gene (*β-actin*) were designed and synthesized by Sangong (Shanghai, China). The primers of genes are shown in Table 1. qRT-PCR was performed in LightCycle 480 II instrument (Roche, Switzerland) using THUNDERBIRD SYBR QPCR Mix (TOYOBO, Japan). The qRT-PCR reaction procedure was: 95 °C for 60 s, followed by 40 cycles at 95 °C for 15 s and 60 °C for 60 s. The CT values were recorded, and then the relative mRNA levels of target genes were calculated according to the 2 ^-ΔΔCT^ method.

**Table 1:** Primer sequences used for the study.

Gene	Gene Reference Sequence	Primer Sequences (5′-3′)
*β-actin*	NM_205518.1	F: CACCACAGCCGAGAGAGAAAT
		R: TGACCATCAGGGAGTTCATAGC
*CIRP*	NM_001031347	F: TCAGAGAACAGATCCCGAGGC
		R: ACCTCCTCTGGAGAAGCCAC
*TRPM8*	NM_001007082.1	F: AGTCCAAAGGGGCCTGGATT
		R: GCCACCACACTCTCCTCAGA
*IκB-α*	NM_001001472.2	F: ATGCTCAGCGCCCAAC
		R: CTTCTCACAGAAACCTCTGC
*P65*	NM_205129	F: GTGTGAAGAAACGGGAACTG
		R: GGCACGGTTGTCATAGATGG
*ERK*	NM_204150	F: CATTCAGCCAATGTGCTTCATCGC
		R: GCAACACGAGCCAGTCCGAAG
*JNK*	XM_001277554.1	F: GTGTGTCCCAGAGGTCAACGAAC
		R: AGCCGAGGTACAGCCAGTCATAG
*P38*	XM_001232615.3	F: CAGATGACGAACCAGTGGCAGAC
		R: CGGTGGCACAAAGCTGATTACTTC
*iNOS*	NM_204961.1	F: CCTGGAGGTCCTGGAAGAGT
		R: CCTGGGTTTCAGAAGTGGC
*COX-2*	NM_001167719.1	F: TGTCCTTTCACTGCTTTCCA
		R: TTCCATTGCTGTGTTTGAGGT
*TNF-α*	AY765397.1	F: GCCCTTCCTGTAACCAGATG
		R: ACACGACAGCCAAGTCAACG
*IL-1β*	NM_204524.1	F: ACTGGGCATCAAGGGCTA
		R: GGTAGAAGATGAAGCGGGTC
*IL-10*	EF554720.1	F: GCTGTCACCGCTTCTTCACCT
		R: GGCTCACTTCCTCCTCCTCATC
*IL-4*	NM_001007079.1	F: AGACAAATAACAAAACTGAGC
		R: TTGGTGGAAGAAGGTACG
*IL-6*	NM_204628.1	F: AAATCCCTCCTCGCCAATCT
		R: CCCTCACGGTCTTCTCCATAAA
*IFN-γ*	NM_205149	F: GAACTGGACAGGGAGAAATGAGA
		R: ACGCCATCAGGAAGGTTGTT

### Western Blot Analysis

A small piece of heart tissue was taken and divided into two parts. One part was added with 1mL of Western and IP cell lysate (Biosharp, Beijing, China) and 10 μ L PMSF (Biosharp, Beijing, China), and the other part was added with 1mL of Western and IP cell lysate, 5 μL PMSF and 5 μL phosphatase inhibitor (Biosharp, Beijing, China). The protein was determined using the BCA protein detection kit (Biosharp, Beijing, China) on the microplate reader (ALLSHENG, Hangzhou, China). The protein concentration was adjusted to a uniform concentration of 5 μg/μL by adding PBS and 5x Protein Loading Buffer. A total of 10 μL protein (50 μg/condition) were fractionated by 10% SDS-PAGE gel. Then, the protein was transferred to the nitrocellulose membrane using semi-dry transfer instrument (Liuyi, Beijing, China). The nitrocellulose membrane was sealed in 5% western block solution at room temperature for 2 h, and then washed with 1 x TBST. The blots were immersed in the primary antibodies at 4 °C overnight. After washing with 1 x TBST, the blots were immersed in the HRP goat anti-rabbit IgG at room temperature for 1 h. The specific dilution ratio of the primary antibodies and IgG is shown in the table S1. Finally, the imaging was performed on the chemiluminescence imaging system (GeneGnomeXRQ, UK) using the ECL chemiluminescence kit (Biosharp, Beijing, China). The gray scale (**IOD**) of blots was analyzed by the Image J software (Media Cybernetic, MD). The levels of the target protein were expressed as the IOD ratio of target protein to β-actin.

### Statistics Analysis

All data were analyzed using IBM SPSS 21.0 software (IBM, Armonk, NY). Normal distribution of data was analyzed by Kolmogorov-Smirnov test. The significant difference between CC and CS was analyzed by independent sample t-test. The results were expressed as mean ± standard deviation (**SD**). *P* value ≤ 0.05 indicates significant difference. *P* value ≤ 0.01 indicates highly significant difference.

## RESULTS

### Effects of IMCS on Histopathology

**Figure 2:**
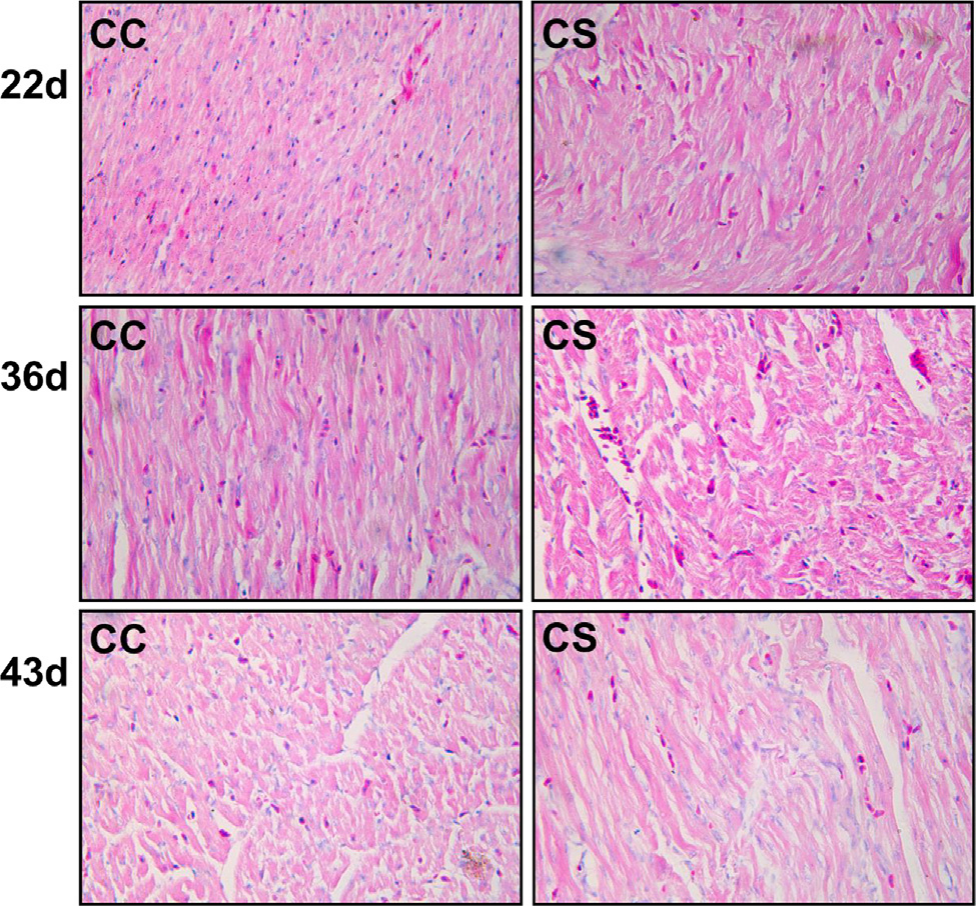
Effects of IMCS on histopathology. The magnification is 400 × .

The histopathological analysis of hearts is shown in Figure 2. IMCS for one week (22 d), the morphology of myocardial cells in the CC and CS groups was normal, with neat and tight arrangement, and there was no infiltration of inflammatory cells. When IMCS ended (36 d), the cells in the CC group were arranged neatly. A low number of inflammatory cells in the CS group. On d 43, the structure of myocardial cells in the CC group was intact and normal. The inflammatory cells in the CS group basically disappeared.

### Effects of IMCS on the Levels of CIRP and Cytokines in the Serum

**Figure 3:**
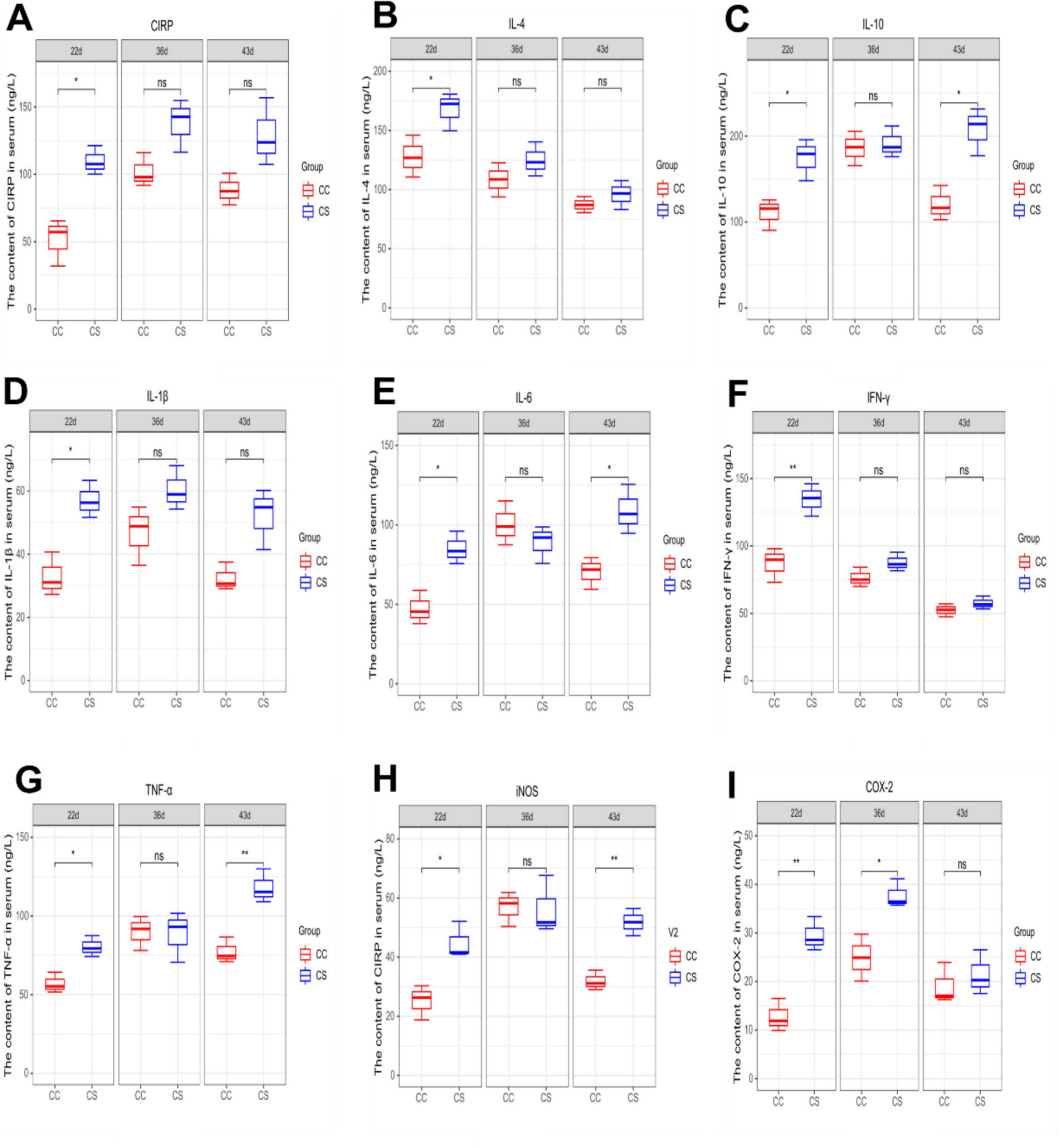
Effects of IMCS on the levels of CIRP and cytokines in the serum. A. CIRP, B. IL-4, C. IL-10, D. IL-1β, E. IL-6, F. IFN-γ, G. TNF-α, H. iNOS, I. COX-2. Values are means ± SD. * *P* ≤ 0.05, ** *P* ≤ 0.01, “ns” means not significant.

In Figure 3 A, on d 22, the CIRP level in the CS group was significantly higher than that in the CC group (*P* ≤ 0.05). There was no significant difference between the two groups on d 36 and 43 (*P* > 0.05).

The results of cytokines levels in serum are shown in Figures 3B to 3I, on d 22, the levels of all cytokines in the CS group were significantly higher than those in the CC group (*P* ≤ 0.05), especially the IFN-γ and COX-2 (*P* ≤ 0.01). On d 36, the level of COX-2 in the CS group was still higher than that in the CC group (*P* ≤ 0.05), while other cytokines levels were not significantly different between the CC and CS groups (*P* > 0.05). On d 43, the contents of IL-10, TNF-α and iNOS in CS group were significantly higher than those in the CC group (*P* ≤ 0.05), but no significant difference was observed in the remaining cytokines between the two groups (*P* > 0.05).

### Effects of IMCS on the mRNA Levels of CIRP and TRPM8, and the Protein Level of CIRP in the Hearts

**Figure 4:**
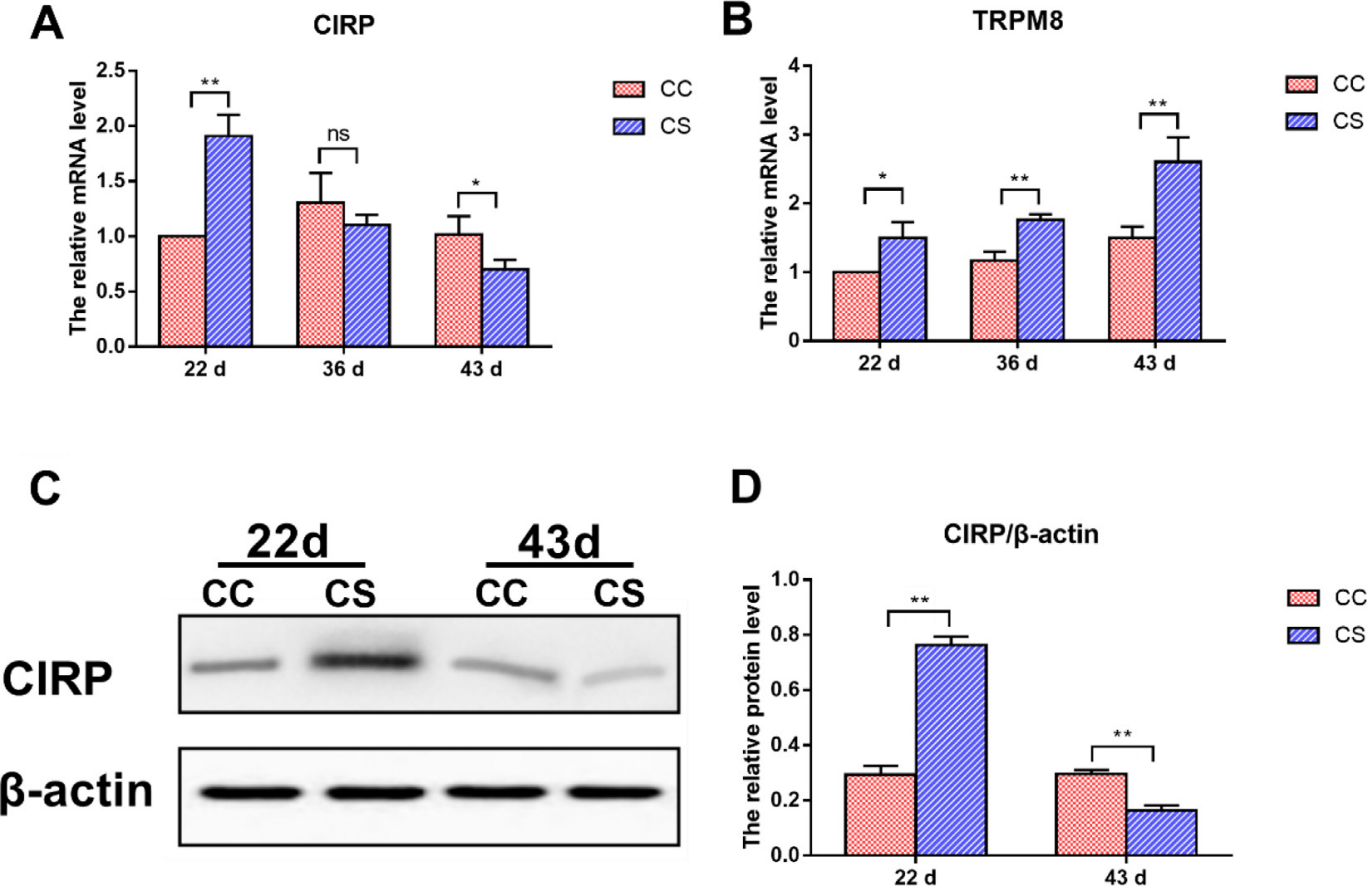
Effects of IMCS on the expression levels of CIRP and TRPM8 in the hearts. A-B. mRNA levels of *CIRP* and *TRPM8*, C-D. protein levels of CIRP. Values are means ± SD. * *P* ≤ 0.05, ** *P* ≤ 0.01, “ns” means not significant.

The mRNA levels of *CIRP* and *TRPM8* are shown in Figures 4A and 4B. On d 22, the mRNA levels of *CIRP* and *TRPM8* in the CS group were significantly higher than those in the CC group (*P* ≤ 0.05). When the IMCS ended (36 d), the *TRPM8* level was still upregulated in the CS group as compared to the CC group (*P* ≤ 0.01), the *CIRP* mRNA level was downregulated, but the difference was not significantly between the 2 groups (*P* > 0.05). On d 43, compared to the CC group, a significant increase in the mRNA level of *TRPM8* was observed in the CS group (*P* ≤ 0.01), while a significant decrease in the *CIRP* mRNA level was observed in the CS group (*P* ≤ 0.05).

The results of CIRP protein level are shown in Figures 4C and 4D. The protein level of CIRP in the CS group was significantly higher than that in the CC group on day 22 (*P* ≤ 0.01). However, when the IMCS ended for one week (43 d), the protein level of CIRP was downregulated in the CS group compared to the CC group (*P* ≤ 0.01).

### Effects of IMCS on the mRNA and Protein Levels of Key Molecules in the NF-κB Pathway

**Figure 5:**
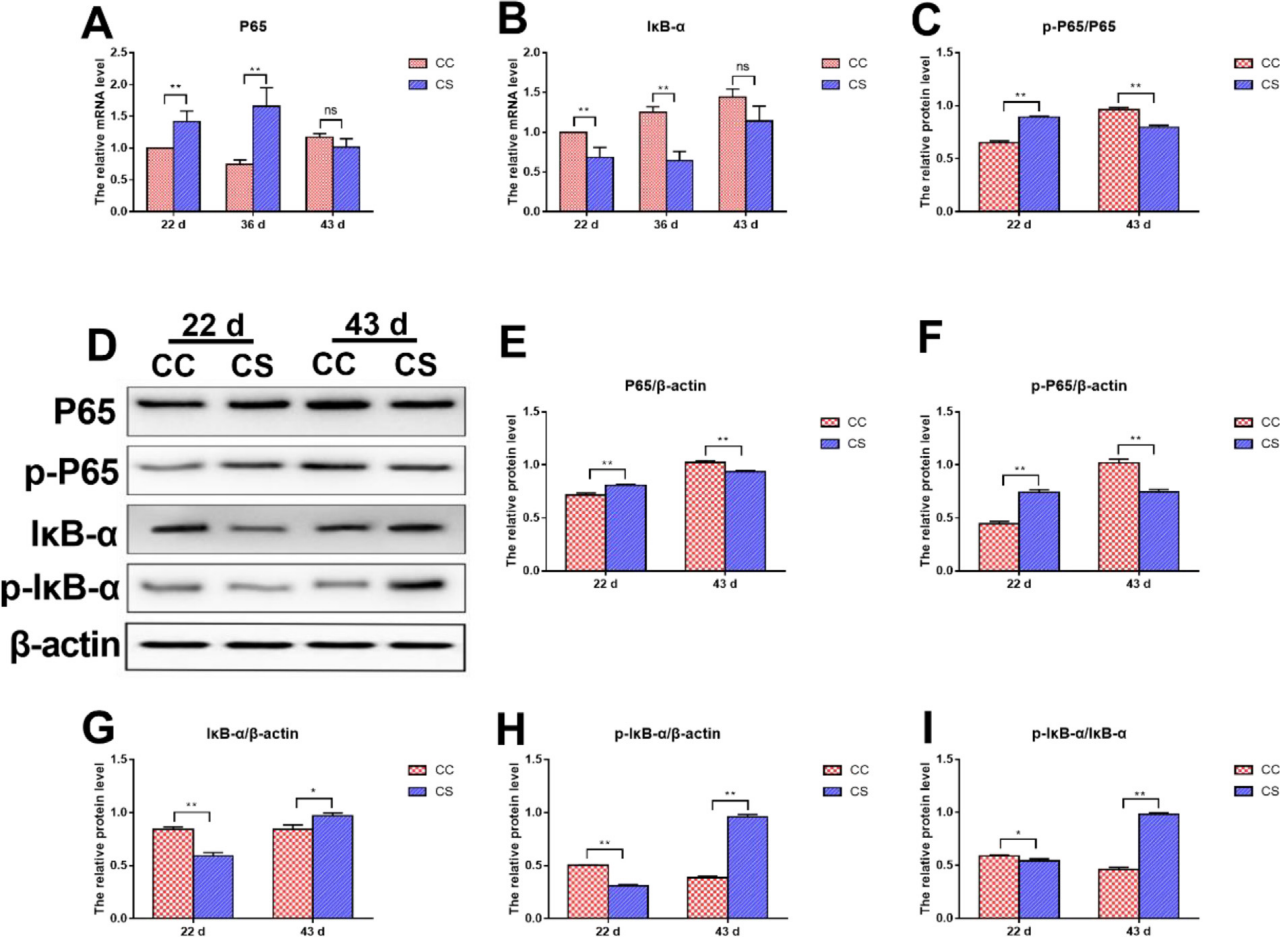
Effects of IMCS on the expression levels of key molecules in the NF-κB pathway in the hearts. A-B. mRNA levels of *P65* and *IκB-α*, C-I. protein levels of P65 and IκB-α. Values are means ± SD. * *P* ≤ 0.05, ** *P* ≤ 0.01, “ns” means not significant.

As shown in Figures 5A and 5B, on d 22 and 36, the mRNA level of *P65* in the CS group was significantly higher than that in the CC group (*P* ≤ 0.01), while that of *IκB*-α was significantly lower in the CS than that in the CC group (*P* ≤ 0.01). When the IMCS ended for 1 wk (on d 43), there was no significant difference in the mRNA levels of *P65* and *IκB-α* between the 2 groups (*P* > 0.05).

The protein levels are shown in Figures 5C to 5I, compared to the CC group, the protein levels of phosphorylated and non-phosphorylated P65 in the CS group were significantly upregulated (*P* ≤ 0.01), but those of IκB-α in the CS group were significantly downregulated on day 22 (*P* ≤ 0.01). In addition, the ratio of p-P65 to P65 in the CS group was significantly higher than that in the CC group (*P* ≤ 0.01), while the ratio of p-IκB-α to IκB-α in the CS group was significantly lower than that in the CC group (*P* ≤ 0.05). The protein levels on day 43 were opposite to that on day 22. Specifically, compared to the CC group, the protein levels of p-P65, P65, and the ratio of p-P65 to P65 in the CS group were significantly lower (*P* ≤ 0.01), those of IκB-α in the CS group were significantly higher (*P* ≤ 0.05).

### Effects of IMCS on the mRNA and Protein Levels of Key Molecules in the MAPK Pathway

**Figure 6:**
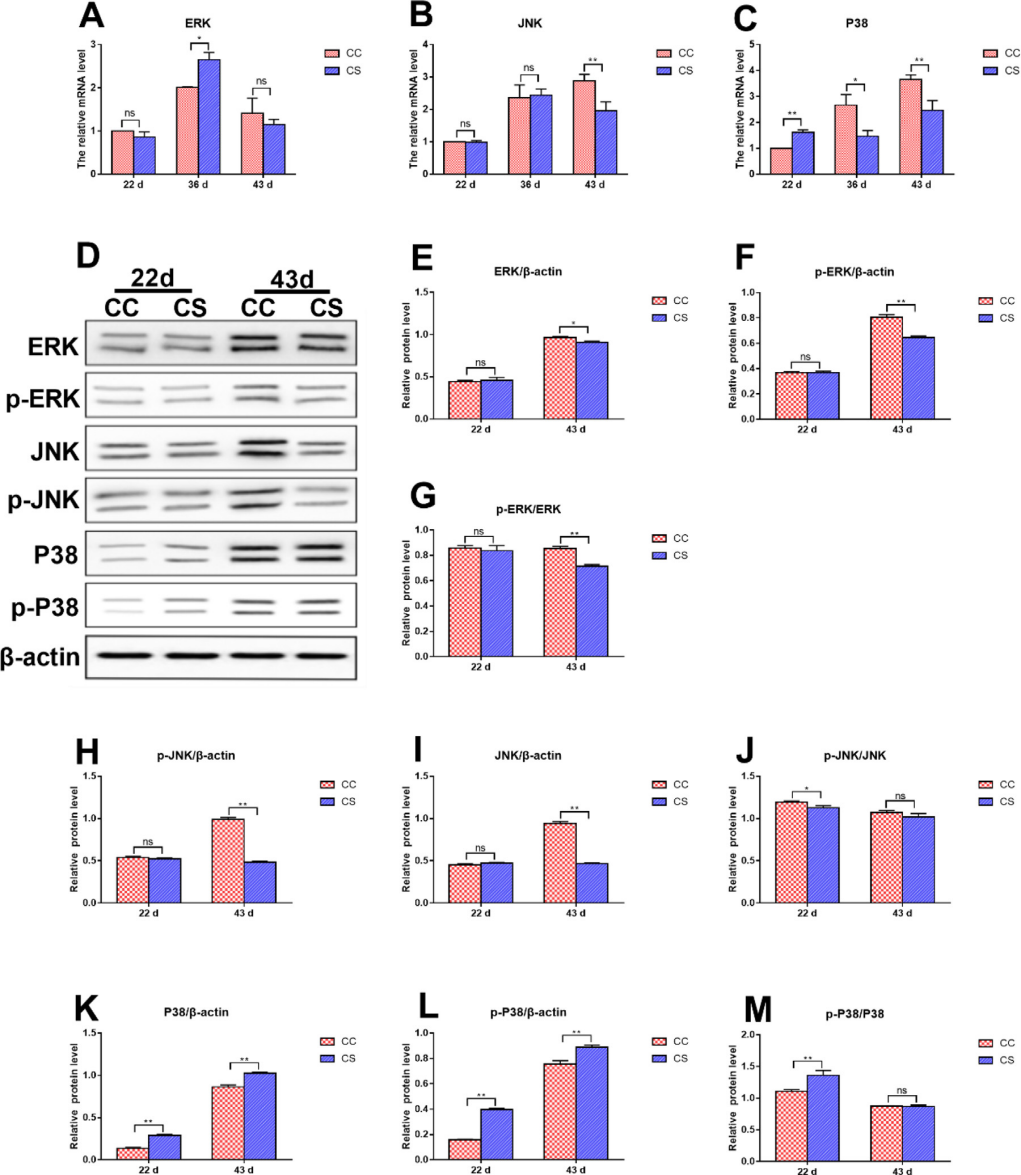
Effects of IMCS on the expression levels of key molecules in the MAPK pathway in the hearts. A-C. mRNA levels of *ERK, JNK* and *P38*, D-M. protein levels of ERK, JNK and P38. Values are means ± SD. * *P* ≤ 0.05, ** *P* ≤ 0.01, “ns” means not significant.

As shown in Figures 6A to 6C, on day 22, a significantly higher mRNA level of *P38* was found in the CS group as compared to the CC group (*P* ≤ 0.01), there was no significant difference in *ERK* and *JNK* mRNA levels between the two groups (*P* > 0.05). On d 36, the mRNA level of *ERK* in the CS group was significantly higher than that in the CC group (*P* ≤ 0.05), but that of *P38* in the CS group was significantly lower than that in the CC group (*P* ≤ 0.05). The *JNK* mRNA level was not different significantly between the 2 groups (*P* > 0.05). On d 43, compared to the CC group, the mRNA levels of *JNK* and *P38* were downregulated (*P* ≤ 0.01), while no significant difference was observed on *ERK* mRNA level (*P* > 0.05).

As shown in Figures 6D to 6M, on d 22, a significantly higher protein levels of P38 and p-P38 in the CS group was found in comparison with the CC group (*P* ≤ 0.01). The ratio of p-P38 to P38 in the CS group was increased significantly (*P* ≤ 0.01), but the ratio of p-JNK to JNK in the CS group was decreased significantly (*P* ≤ 0.05) compared to the CC group. On d 43, the protein levels of ERK, p-ERK, JNK and p-JNK in the CS group were significantly lower than those in the CC group (*P* ≤ 0.05), but those of the P38 and p-P38 in the CS group were significantly higher than those in the CC group (*P* ≤ 0.01). The ratio of p-ERK to ERK was significantly decreased (*P* ≤ 0.01), there was no significant difference on the ratio of p-JNK to JNK, p-P38 to P38 (*P* > 0.05).

### Effects of IMCS on the mRNA and Protein Levels of Cytokines in Hearts

**Figure 7:**
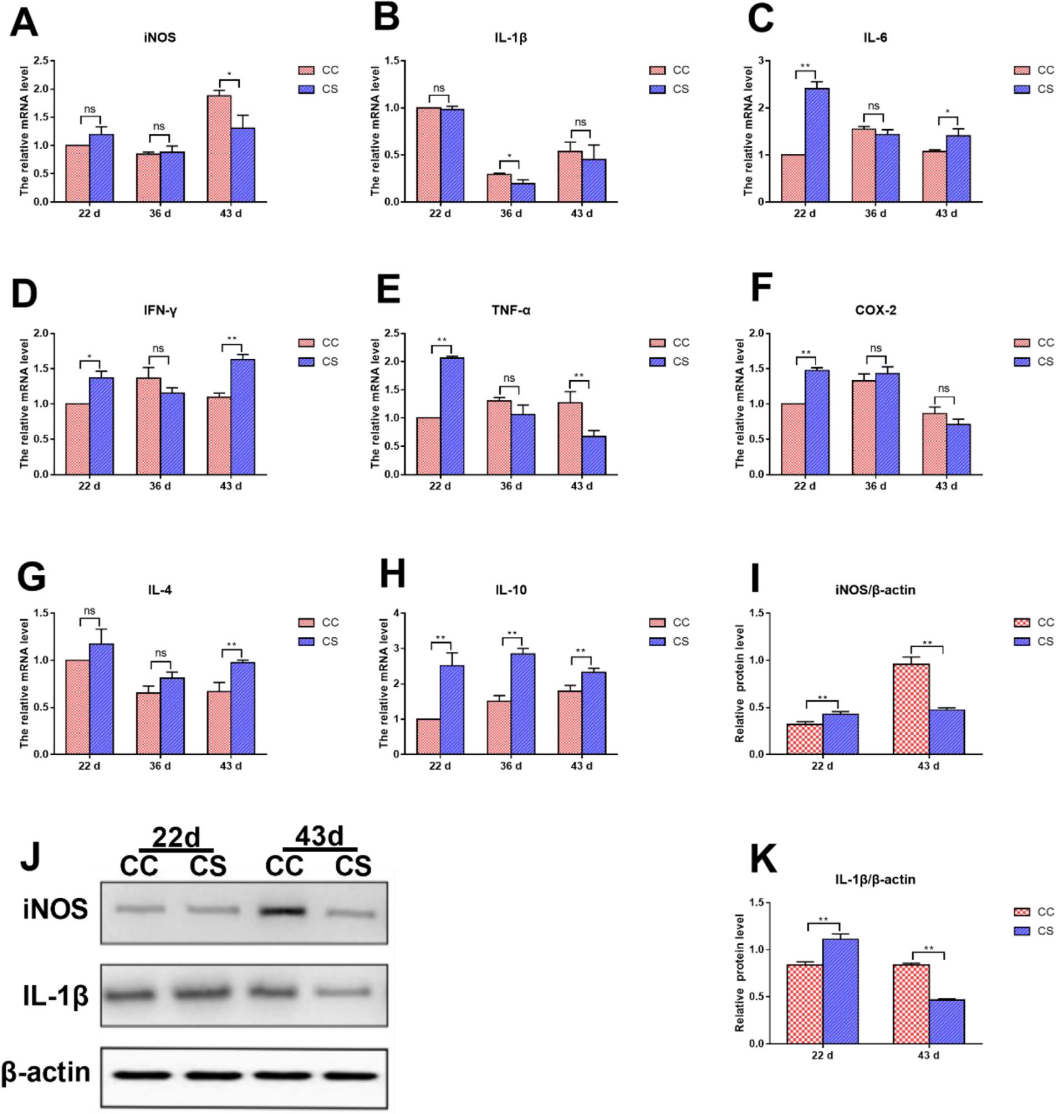
Effects of IMCS on the expression levels of cytokines in the hearts. A-H. mRNA levels of *iNOS, IL-1β, IL-6, IFN-γ, TNF-α, COX-2, IL-4* and *IL-10*, I-K protein levels of iNOS and IL-1β. Values are means ± SD. * *P* ≤ 0.05, ** *P* ≤ 0.01, “ns” means not significant.

The results of the mRNA levels of cytokines are shown in Figures 7A to 7H. On d 22, the mRNA levels of *IL-6, IFN-γ, TNF-α, COX-2* and *IL-10* in the CS group were significantly higher than those in the CC group (*P* ≤ 0.05). The mRNA levels of other cytokines in the CS group were not different from those in the CC group (*P* > 0.05). On d 36, the *IL-10* mRNA level in the CS group was increased significantly (*P* ≤ 0.01), while the *IL-1β* mRNA level in the CS group was decreased significantly compared to the CC group (*P* ≤ 0.05). No significant difference was found in the mRNA levels of other cytokines between the CC and CS groups (*P* > 0.05). On d 43, compared to the CC group, the mRNA levels of *IL-4, IL-6, IL-10* and *IFN-γ* in the CS group were significantly upregulated (*P* ≤ 0.05), while the mRNA levels of *iNOS* and *TNF-α* were significantly downregulated (*P* ≤ 0.05). The mRNA levels of *IL-1β* and *COX-2* had no significant difference between the two groups (*P* > 0.05).

As shown in Figures 7I to 7K, compared to the CC group, the protein levels of iNOS and IL-1β in the CS group were significantly upregulated on d 22 (*P* ≤ 0.01), but those in the CS group were significantly downregulated on d 43 (*P* ≤ 0.01).

## DISCUSSION

When the body is exposed to a low-temperature environment, CIRP is immediately activated and previous studies have shown that CIRP is one of the stress proteins produced by mild hypothermia (Zhang et al., 2015). This can help the cells and the body to quickly adapt to the cold environment. The expression level of CIRP in neural stem cells increased significantly after 24 and 48 hours of cold stimulation at 32 °C in vitro (Saito et al., 2010). Conversely, under heat treatment (39 °C and 42 °C), its expression level is significantly decreased (Nishiyama et al., 1998). On the other hand, TRPM8 participates in temperature regulation and plays an important role in the perception of low temperature. What is more important, TRPM8 also participates in immune protection at low temperatures and is closely related to temperature adaptation (Bautista et al., 2007; Cymbalyuk et al., 2011; Zhang et al., 2016). Research has shown that during cold stress, TRPM8 is upregulated in the hypothalamus of mice and has a negative regulatory effect on TNF-α by inhibiting the activation of the NF-κB pathway. Also, the knockdown of the expression of TRPM8 in cells results in an increased expression level of TNF-α (Wang et al., 2017). Similar to the above results, the present study showed that in the early stage of IMCS (22 d), cold stimulation upregulated the expression levels of CIRP and *TRPM8* in the heart, as well as the content of CIRP in the serum. This could activate the corresponding pathways and innate immunity to help the body adapt to the cold environment (Liu et al., 2018). One week after the IMCS training (43 d), the expression level of CIRP in the CS group was downregulated, and the CIRP content in the serum gradually returned to normal. This may prevent the excessive release of CIRP into the extracellular environment and furthermore, avoid the inflammatory reactions. However, *TRPM8* was still maintained at a high level, indicating that TRPM8 played an important role in establishing and maintaining cold adaptation.

The NF-κB protein is one of the main regulatory factors for inflammation and immune homeostasis. It is quickly activated by environmental stimuli to initiate immune regulation and enhance resistance to stress (Wang et al., 2018). Also, MAPK, mainly including the ERK, P38 and JNK pathways, can be activated by various forms of stimulation and play an important regulatory role in the occurrence and development of inflammation, and maintenance of immune balance (Huizinga et al., 2006; Zhou et al., 2018). In the present study, during the IMCS (22-36 d), the NF-κB, ERK, and P38 pathways were activated in the heart, and the expression levels of anti-inflammatory factors (IL4 and IL-10) were upregulated. Moreover, pro-inflammatory factors (COX-2, TNF-α, iNOS, IFN-γ, IL-1 β and IL6) were also upregulated in the early stages of cold training, but the expression levels returned to normal on day 36. Furthermore, H&E staining revealed a low number of inflammatory cells in the CS group, and heart's structure returned to normal after the cold stimulation ended. These findings suggest that the broilers gradually adapted to the low-temperature environment during cold training, and this cold training strategy did not cause damage to the cardiac structure or immune function. When the IMCS ceased for one week (43 d), the key genes in the NF-κB, ERK and JNK pathways were downregulated, and the key genes expressed in the P38 pathway were upregulated, leading to the downregulation of pro-inflammatory factors (iNOS, IL-1β, COX-2 and TNF-α). Conversely, the expression levels of anti-inflammatory cytokines (IL-4 and IL-10) were upregulated continuously, indicating an improvement in the anti-inflammatory ability and immune function of the broilers. Similarly, heat stress can enhance the anti-inflammatory ability of mice by activating the JNK and P38 pathways (Dryer et al., 2012). Exposure to a 35 °C thermal environment can activate the MAPK pathway in the heart and skeletal muscles of broilers, thus reducing the cell apoptosis induced by heat stress (Srikanth et al., 2019). Liu et al. showed that during the early stages of cold stimulation training, the expression levels of pro-inflammatory factors such as IL-6, IL-8, and IFN-γ increased in the bursa of broilers. This can activate both innate and adaptive immunity, and help the broilers adapt to the cold. However, after the establishment of cold adaptation, the expression levels of the pro-inflammatory factors decreased (Liu et al., 2020). Similarly, cold stimulation at 3 °C below normal feeding temperature did not damage the immune balance of broilers and enhanced their immune function to a certain extent (Wei et al., 2018; Xing et al., 2023; Zhang et al., 2023). Thus, changes in the expression levels of CIRP and TRPM8 play a crucial role in regulating the NF-κB and MAPK signaling pathways. In the early stages of cold stimulation, IMCS can activate the NF-κB, ERK, and P38 pathways by upregulating the CIRP and TRPM8 levels, and then upregulating the immune cytokines without disrupting the immune balance. Finally, the immune regulatory function of broilers can be activated to help them adapt to low-temperature environments. After IMCS ended, CIRP levels in the heart and serum are decreased, but TRPM8 remained high, to inhibit the NF-κB, ERK, and JNK pathways, which can prevent the excessive release of inflammatory cytokines, thereby enhancing the anti-inflammatory ability of broilers.

## CONCLUSIONS

In the early stages of cold stimulation, CIRP and TRPM8 co-regulate NF-κB and MAPK signaling pathways, which can activate the immune function of broilers to resist cold environments. With the prolongation of IMCS, the expression of CIRP is downregulated to avoid inflammatory reactions, which is caused by excessive release of CIRP into the blood. Meanwhile, the continued increased expression of TRPM8 inhibits the persistent activation of the NF-κB and MAPK pathways, and the excessive release of inflammatory factors. These changes in the expression levels of CIRP and TRPM8 can maintain immune function balance and provide immune protection to broilers. The findings of this work contribute to the understanding of cold adaptation in broilers and its relevance to animal husbandry.

## DISCLOSURES

The authors declare no conflicts of interest.

## Appendix Supplementary materials

Image, application 1

## Footnotes

Supplementary material associated with this article can be found in the online version at doi:10.1016/j.psj.2024.103984.
