# Supplementary material for: Intermittent and mild cold stimulation enhances immune function of broilers via co-regulation of CIRP and TRPM8 on NF-κB and MAPK signaling pathways
Source: Poult Sci. 2024 Jun 17;103(9):103984. doi: 10.1016/j.psj.2024.103984 (PMC11296020; doi:10.1016/j.psj.2024.103984)
Supplement: Supplementary file 1 [file mmc1.docx]

**Appendix. Supplementary material:**

Table S1 The dilution ratio of antibodies

| Antibody | Dilution Ratio | Manufacturer |
| --- | --- | --- |
| CIRP | 1:1000 | Bioss, China |
| IKB-α | 1:500 | Wanleibio, China |
| p-IKB-α | 1:500 | Wanleibio, China |
| P65 | 1:500 | Wanleibio, China |
| p-P65 | 1:500 | Wanleibio, China |
| ERK | 1:2000 | Cell Signaling, America |
| p-ERK | 1:1000 | Cell Signaling, America |
| JNK | 1:1000 | Cell Signaling, America |
| p-JNK | 1:1000 | Cell Signaling, America |
| P38 | 1:1000 | Cell Signaling, America |
| p-P38 | 1:1000 | Cell Signaling, America |
| iNOS | 1:800 | Wanleibio, China |
| IL-1β | 1:500 | Wanleibio, China |
| β-actin | 1:4000 | Sangong China |
| IgG | 1:40000 | Sangong, China |
